# Supplementary material for: The EYA3 tyrosine phosphatase activity promotes pulmonary vascular remodeling in pulmonary arterial hypertension
Source: Nat Commun. 2019 Sep 12;10:4143. doi: 10.1038/s41467-019-12226-1 (PMC6742632; doi:10.1038/s41467-019-12226-1)
Supplement: Supplementary file 1 — Supplementary Information [file 41467_2019_12226_MOESM1_ESM.pdf]

The EYA3 Tyrosine Phosphatase Activity Promotes Pulmonary Vascular Remodeling in Pulmonary Arterial Hypertension

Wang *et al*

Supplementary Information (5 figures, 1 table)

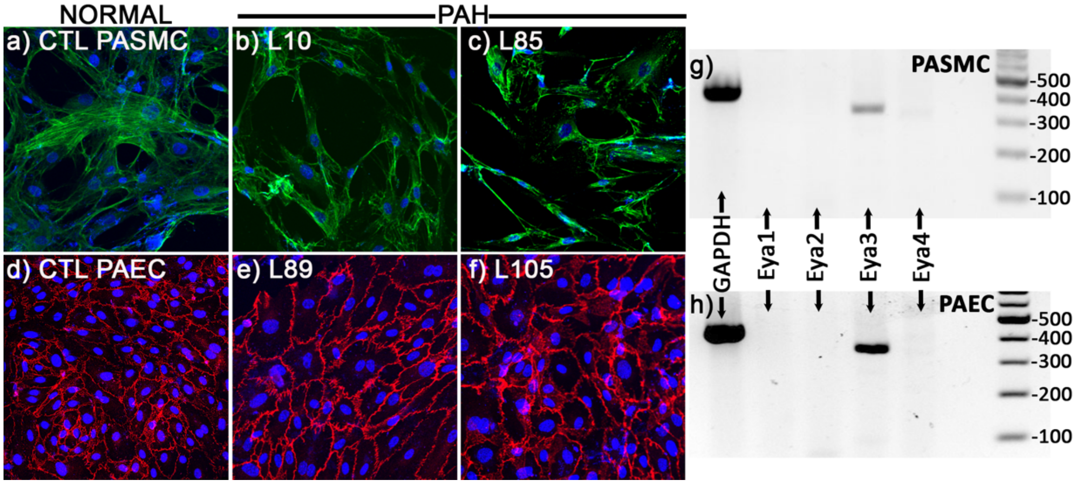

Supplementary Figure 1. Characterization of human pulmonary vascular cells.

a, b, c) Characterization of pulmonary arterial smooth muscle cells (PASMC). PASMC (CTL (normal donor), L10 (PAH) and P85 (PAH) stained with anti α-SMA and counter-stained with DAPI.

d, e, f) Characterization of pulmonary arterial endothelial cells (PAEC). PAEC (CTL (normal donor), L89 (PAH) and P105 (PAH) stained with anti VE-cadherin and counter-stained with DAPI.

g) **RT-PCR analysis on mRNA isolated from primary human pulmonary arterial smooth muscle cells (PASMC)**. Products after 30 cycles were run on a 1.5% agarose gel. Expected bands were 547 bp for *Eya1*, 132 bp for *Eya2*, 341 bp for *Eya3*, 397 bp for *Eya4*, and 453 bp for GAPDH (control). Only E*ya3* transcript was detected. 100 base-pair molecular weight marker is shown in the right lane.

h) **RT-PCR analysis on mRNA isolated from primary human pulmonary arterial endothelial cells (PAEC)**. Products after 30 cycles were run on a 1.5% agarose gel. Expected bands were 547 bp for *Eya1*, 132 bp for *Eya2*, 341 bp for *Eya3*, 397 bp for *Eya4*, and 453 bp for GAPDH (control). Only E*ya3* transcript was detected. 100 base-pair molecular weight marker is shown in the right lane.

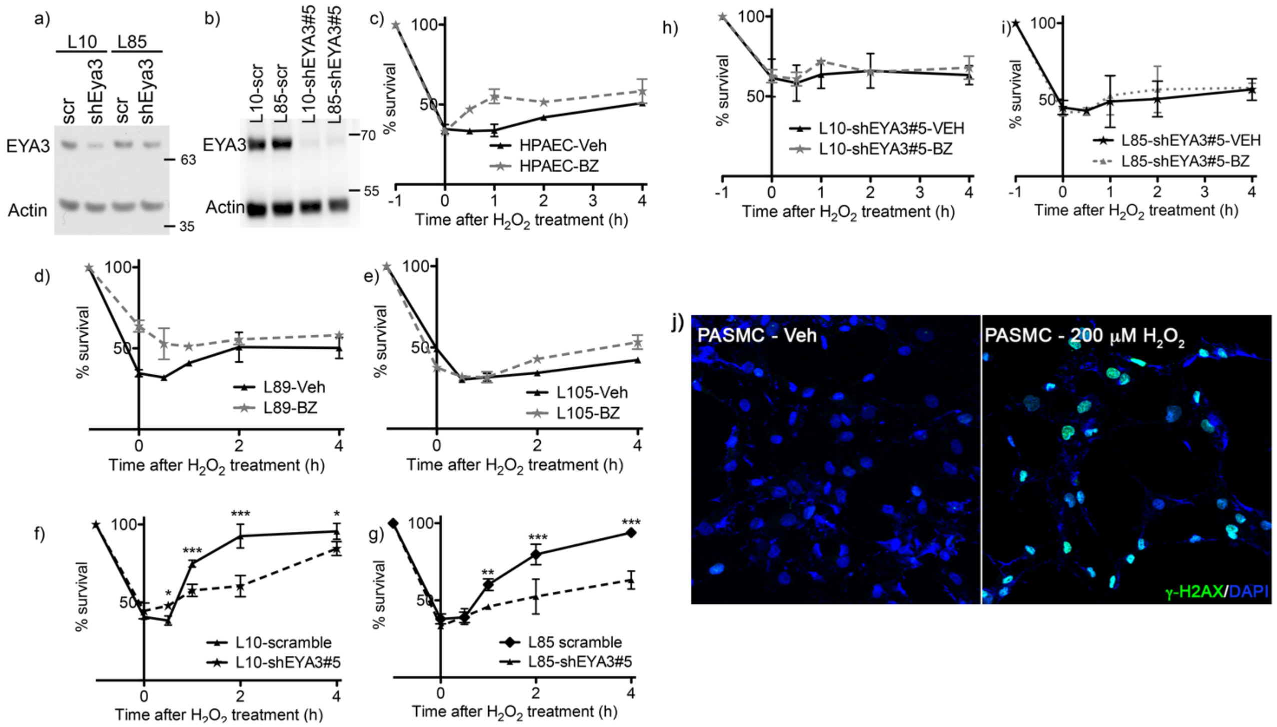

Supplementary Figure 2. Survival of pulmonary vascular cells after oxidative stress.

a) Western blots showing EYA3 protein levels in EYA3 L10 and L85 cells used in Fig. 2.

b) Western blots showing EYA3 protein levels with an additional shEYA3 construct.

c) EYA-PTP inhibition with Benzarone has no effect on the susceptibility of normal HPAEC cells to H_2O_2 treatment. In each experiment (c-i) cells were treated with 200 μM H_2O_2 for 1 hour, then withdrawn and cells allowed to recover in normal culture medium. Percentage of viable cells were monitored using the WST-8 cell viability assay and are plotted versus time (x-axis). -1h indicates the start of H_2O_2 treatment. H_2O_2 was withdrawn at 0h. Representative data shown as the mean ± SD; statistical significance was determined using two-way ANOVA and Bonferroni's post-test, *** $P<0.0001$, * $P<0.05$.

d – e) EYA-PTP inhibition with BZ has no effect on PAH-PAEC L89 (d) or L105 (e) survival after H_2O_2 treatment.

f – g) Stable knockdown of EYA3 in PAH-PASMC L10 (f) or L85 (g) cells increases their susceptibility to H_2O_2 treatment. This experiment reproduces the outcome presented in Fig. 2 using a different shRNA construct

h, i) EYA-PTP inhibition with Benzarone has no effect on the susceptibility of PAH-PASMC L10-shEYA3 (h) or L85-shEYA3 (i) cells to H_2O_2 treatment. This experiment reproduces the outcome presented in Fig. 2 using a different shRNA construct.

j) 200 μM H_2O_2 treatment for 1 hour induces DNA damage response as indicated by γ-H2AX staining (green).

Source Data are provided as a Source Data file.

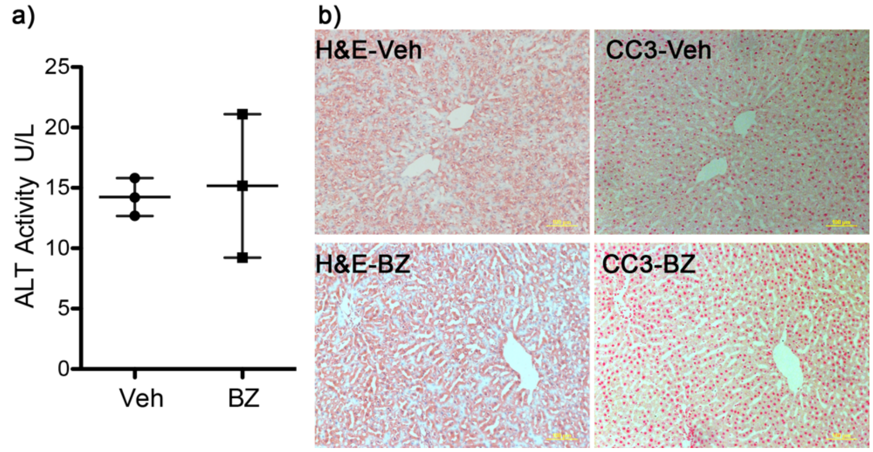

Supplementary Figure 3. Evaluation of Benzarone toxicity.

a) Rats were treated with either Vehicle or BZ using the dosing schedule (treatment arm) described in Figure 3. Levels of Alanine Aminotransferase (ALT) in the serum was measured using the commercial Alanine Transaminase Activity Assay Kit (Abcam ab105134) as a measure of hepato-toxicity. In this assay ALT transfers an amino group from alanine to α-ketoglutarate leading to the formation of pyruvate and glutamate. The levels of pyruvate were detected in a colorimetric assay (ODmax = 570 nm) and compared to a standard curve. There was no significant difference in the levels of ALT activity between vehicle and BZ treated rats. Data represent 3 animals in each group and are plotted as the mean and SD.

b) Serial sections of rat livers treated with either vehicle or BZ with the same dosing schedule as the treatment arm in Fig. 3 were stained with H&E and cleaved caspase 3 (CC3). Slides were examined by a blinded pathologist and no histo-pathological evidence of hepatotoxicity was reported. Representative H&E and CC3 stained sections are shown.

Source Data are provided as a Source Data file.

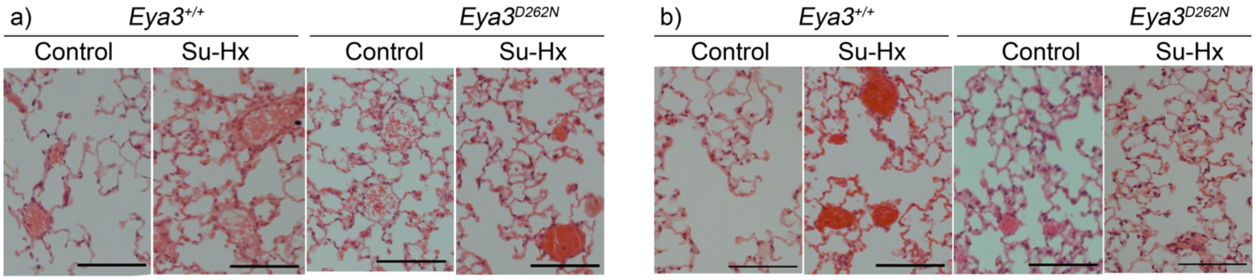

Supplementary Figure 4. H& E images of serial sections indicating the blood vessels imaged in Figure 6.

a) Images correspond to Fig. 6d.
b) Images correspond to Fig. 6e.

Scale bar is 100 μm.

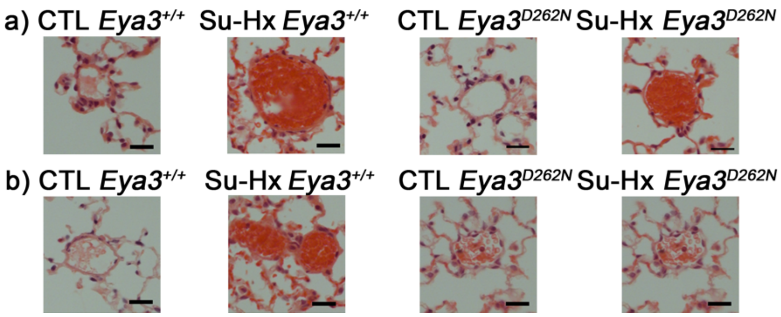

Supplementary Figure 5. H& E staining of serial sections from those in Figure 8 indicating the blood vessels imaged.

a) Images correspond to Fig. 8a.
b) Images correspond to Fig. 8b.

Scale bar is 20 μm.

Supplementary Table 1. iPAH cells obtained from the Pulmonary Hypertension Breakthrough Initiative (PHBI)

PHBI ID #	Penn ID	Gender	Disease	Type of Cells	ID in this manuscript
PHBI-CC-005	10	Female	IPAH	SMC	L10
PHBI-ST-020	85	Female	IPAH	SMC	L85
PHBI-VA-011	105	Female	IPAH	EC III	L105
PHBI-CC-013	89	Female	IPAH	EC III	L89
