# Supplementary material for: The EYA3 tyrosine phosphatase activity promotes pulmonary vascular remodeling in pulmonary arterial hypertension
Source: Nat Commun. 2019 Sep 12;10:4143. doi: 10.1038/s41467-019-12226-1 (PMC6742632; doi:10.1038/s41467-019-12226-1)
Supplement: Supplementary file 3 — Reporting Summary [file 41467_2019_12226_MOESM3_ESM.pdf]

natureresearch

Corresponding author(s): Rashmi Hegde

Last updated by author(s): Aug 27, 2019

Reporting Summary

Nature Research wishes to improve the reproducibility of the work that we publish. This form provides structure for consistency and transparency in reporting. For further information on Nature Research policies, see Authors & Referees and the Editorial Policy Checklist.

Statistics

For all statistical analyses, confirm that the following items are present in the figure legend, table legend, main text, or Methods section.

n/a	Confirmed	
☐	☒	The exact sample size (*n*) for each experimental group/condition, given as a discrete number and unit of measurement
☐	☒	A statement on whether measurements were taken from distinct samples or whether the same sample was measured repeatedly
☐	☒	The statistical test(s) used AND whether they are one- or two-sided<br>*Only common tests should be described solely by name; describe more complex techniques in the Methods section.*
☐	☒	A description of all covariates tested
☒	☐	A description of any assumptions or corrections, such as tests of normality and adjustment for multiple comparisons
☐	☒	A full description of the statistical parameters including central tendency (e.g. means) or other basic estimates (e.g. regression coefficient) AND variation (e.g. standard deviation) or associated estimates of uncertainty (e.g. confidence intervals)
☐	☒	For null hypothesis testing, the test statistic (e.g. *F*, *t*, *r*) with confidence intervals, effect sizes, degrees of freedom and *P* value noted<br>*Give P values as exact values whenever suitable.*
☒	☐	For Bayesian analysis, information on the choice of priors and Markov chain Monte Carlo settings
☒	☐	For hierarchical and complex designs, identification of the appropriate level for tests and full reporting of outcomes
☒	☐	Estimates of effect sizes (e.g. Cohen's *d*, Pearson's *r*), indicating how they were calculated

Our web collection on statistics for biologists contains articles on many of the points above.

Software and code

Policy information about availability of computer code

Data collection	Scisence ADVantage Pressure-Volume control unit, ADV500 System, Transonic, Ithaca NY USA
Data analysis	PRISM5 for Mac OS X V5.0a<br>IMAGEJ 1.60i<br>LabChart Software V7

For manuscripts utilizing custom algorithms or software that are central to the research but not yet described in published literature, software must be made available to editors/reviewers. We strongly encourage code deposition in a community repository (e.g. GitHub). See the Nature Research guidelines for submitting code & software for further information.

Data

Policy information about availability of data

All manuscripts must include a data availability statement. This statement should provide the following information, where applicable:

- Accession codes, unique identifiers, or web links for publicly available datasets
- A list of figures that have associated raw data
- A description of any restrictions on data availability

The authors declare that all data supporting the findings of this study are available within the paper and its supplementary information files and that all data are available from the corresponding author upon reasonable request. The source data underlying Figs 1-8 and Supplementary Figs 2 and 3 are provided as a Source Data file.

Field-specific reporting

Please select the one below that is the best fit for your research. If you are not sure, read the appropriate sections before making your selection.

☒ Life sciences ☐ Behavioural & social sciences ☐ Ecological, evolutionary & environmental sciences

For a reference copy of the document with all sections, see nature.com/documents/nr-reporting-summary-flat.pdf

Life sciences study design

All studies must disclose on these points even when the disclosure is negative.

Sample size	Power analysis was not used since no previous findings on EYA-PTP inhibition in PH models were available to estimate effect size. In rat studies we used a resource equation to obtain an E value between 10 and 20 as recommended (Festing MF, Altman DG. "Guidelines for the design and statistical analysis of experiments using laboratory animals". ILAR J. 2002; 43:244-58). E = total number of animals - total number of groups. Mouse experiments were conducted using litter-mates and were dictated by litter size.
Data exclusions	Mortality in the control PH group was seen in mouse experiments (as reported in the manuscript). Statistical analyses did not include these animals.
Replication	Replication of experiments as reported in the manuscript were successful.
Randomization	Animals were randomly assigned into the treatment groups used.
Blinding	Animals were numbered and either genotype or treatment was not associated with the numbers assigned while the experimenter conducted the PH measurements.

Reporting for specific materials, systems and methods

We require information from authors about some types of materials, experimental systems and methods used in many studies. Here, indicate whether each material, system or method listed is relevant to your study. If you are not sure if a list item applies to your research, read the appropriate section before selecting a response.

Materials & experimental systems

n/a	Involved in the study	
☐	☒	Antibodies
☒	☐	Eukaryotic cell lines
☒	☐	Palaeontology
☐	☒	Animals and other organisms
☐	☒	Human research participants
☒	☐	Clinical data

Methods

n/a	Involved in the study	
☒	☐	ChIP-seq
☒	☐	Flow cytometry
☒	☐	MRI-based neuroimaging

Antibodies

Antibodies used	Goat anti-SM22 Abcam AB10135<br>Alexa-595 conjugated anti-goat secondary antibody (Molecular Probes)<br>rabbit anti-53BP1 (CST #4937)<br>mouse anti-g-H2AX (Millipore 05-636)<br>rabbit anti-Von Willebrand Factor (Abcam AB6994)<br>rabbit anti-Ki67 (Thermo Scientific MA5-14520)<br>Anti-EYA3 (Abcam AB95876)<br>pan anti-actin C4 (Seven Hills Bioreagents)<br>Anti-EYA3 (Proteintech 21196-1-AP)<br>Anti-alpha smooth muscle actin (Thermo Fisher MA5-11547)
Validation	All antibodies used were commercial and validated by the manufacturer.

Animals and other organisms

Policy information about studies involving animals; ARRIVE guidelines recommended for reporting animal research

Laboratory animals	Mice: C57BL/6J from Jackson Laboratories, 8 week-old, both males and females. Transgenic Eya3(D262N) mice generated in the CCHMC facility in a C57BL/6J background, 8 week-old, both sexes used.<br>Rats: 6 - 8 week old Sprague-Dawley rats from Charles River Laboratories.

Wild animals	*Provide details on animals observed in or captured in the field; report species, sex and age where possible. Describe how animals were caught and transported and what happened to captive animals after the study (if killed, explain why and describe method; if released, say where and when) OR state that the study did not involve wild animals.*
Field-collected samples	*For laboratory work with field-collected samples, describe all relevant parameters such as housing, maintenance, temperature, photoperiod and end-of-experiment protocol OR state that the study did not involve samples collected from the field.*
Ethics oversight	All animal protocols were approved by the Cincinnati Children's Hospital Medical Center IACUC (protocol number IACUC2016-0062),

Note that full information on the approval of the study protocol must also be provided in the manuscript.

Human research participants

Policy information about studies involving human research participants

Population characteristics	Human research participants were not directly recruited for this study. They were obtained as de-identified samples from biobanks.
Recruitment	PAEC and PASMC from patients diagnosed with idiopathic PAH were provided by the University of Pennsylvania Cell Center under the Pulmonary Hypertension Breakthrough Initiative (PHBI). De-identified Human lung tissue explants were obtained from the Cincinnati Children's Hospital Medical Center Transplant Service.
Ethics oversight	The use of de-identified human tissue/cells was reviewed by the Cincinnati Children's Hospital Medical Center Institutional Review Board (Study ID:2016-1348) who determined that the studies did not meet the regulatory criteria for research involving human subjects.

Note that full information on the approval of the study protocol must also be provided in the manuscript.
